# A novel potential measurement indicator with objective and quantitative effect for trigeminal neuralgia: fractional anisotropy in MR-DTI

**DOI:** 10.3389/fneur.2024.1453431

**Published:** 2024-12-24

**Authors:** Zhongshuai Ma, Xu Su, Zhengming Wang, Zhijia Wang, Min Cheng, Yu Tian, Chao Du

**Affiliations:** ^1^Department of Neurosurgery, China-Japan Union Hospital of Jilin University and The Third Bethune Hospital of Jilin University, Changchun, China; ^2^Department of Neurosurgery, Xinqiao Hospital, Army Medical University, Chongqing, China; ^3^Department of Trauma Center, China-Japan Union Hospital of Jilin University and The Third Bethune Hospital of Jilin University, Changchun, China; ^4^Department of Radiology, China-Japan Union Hospital of Jilin University and The Third Bethune Hospital of Jilin University, Changchun, China

**Keywords:** pain measurement indicator, trigeminal neuralgia, fractional anisotropy, magnetic resonance diffusion tensor imaging, percutaneous stereotactic radiofrequency rhizotomy

## Abstract

**Objectives:**

To investigate the effect of diffusivity metrics of magnetic resonance diffusion tensor imaging (MR-DTI) in the assessment of treatment effects.

**Methods:**

MR-DTI examination for trigeminal neuralgia (TN) patients and the diffusivity metrics of the trigeminal ganglion (TG) were analyzed. Before and after the percutaneous stereotactic radiofrequency rhizotomy (PSR) operation, the treatment effect was assessed using pain scores and MR-DTI. The correlation between the diffusivity metrics of cranial nerve five (CNV) and visual analog scale (VAS) pain scores before and after treatment in TN patients was explored.

**Results:**

In PSR patients, the fractional anisotropy (FA) of the affected TG is a significantly lower than that of the unaffected side (*p* < 0.01). After PSR, the diffusivity metrics on the bilateral TGs are not significantly different (*p* > 0.05). Following PSR treatment, both of the diffusivity metrics (FA) in the lowest area of the CNV and the VAS scores of TN patients show changes. Furthermore, diffusivity metric (FA) on the lowest area of the CNV preoperative is significantly negatively correlated with the VAS scores (*p* < 0.05).

**Conclusion:**

MR-DTI is capable of investigating the longitudinal changes of FA before and after radiofrequency treatment, and diffusivity metrics could be an independent reliable efficacy indicator for TN.

**Significance:**

The alteration of the diffusivity on TG may be correlated with the effect of radiofrequency treatment.

## 1 Introduction

Trigeminal neuralgia (TN) is a severe neuropathic pain disorder affecting one or more branches of the trigeminal nerve (CNV, the cranial nerve five) (1, 2). Percutaneous stereotactic radiofrequency rhizotomy (PSR) is an effective and minimally invasive therapy for TN, with the treatment target being the trigeminal ganglion (TG) (3–8). Several studies have proposed that the microstructural changes of CNV can be quantified using magnetic resonance diffusion tensor imaging (MR-DTI) to identify focal lesions (5, 9–11). Fractional anisotropy (FA) is the most common diffusivity metric of the MR-DTI, and the diffusivity characteristics of the TG target could guide radiofrequency treatment and directly reflect the alteration of TG in patients with TN (12).

MR-DTI is a quantitative imaging technique based on the principle of the motion of water molecules in neural tissue (13–15). DTI has recently become widely used for the quantification of neuro tracts via tractography non-invasively (10, 16). Tractography can accurately delineate the visual 3D anatomical structures of nerve tracts and their branches, which are not visible on conventional morphological MRI (17, 18). A nerve-specific DTI could assess the nerve fiber, axon, and myelin microstructure (16, 19), and provide a clearer visualization and estimation of neural tract injury at the microscopic level, which is crucial in the treatment and pathogenesis (10, 11, 20, 21).

Recently, DTI studies on TN have focused on the CNV, and studies on the evaluations of treatment outcome in different therapies, such as microvascular decompression (MVD), percutaneous stereotactic radiofrequency rhizotomy (PSR), and gamma knife radiosurgery (GKRS). However, diffusivity on the trigeminal ganglion has rarely been reported in previous studies (22, 23). This study aimed to investigate the effect of diffusivity metrics of MR-DTI in the assessment of treatment effects. It may be beneficial to TN treatment and management in clinical practice.

Visual algology scale (VAS) score is the most common a sensitive assessment of pain intensity in TN. However, VAS scores were easily affected by cognitions, subjective factors, and emotion (24–27). Up to now, there is no objective and quantitative pain assessment indicator in the clinical management of TN. Toward this goal, we explored the correlation between diffusivity metrics of CNV and visual analog scale (VAS) pain scores before and after treatment in TN patients.

## 2 Materials and methods

### 2.1 Study design and participants

This observational study involved 28 TN patients. The TN group was further divided into the operation subgroup and the non-operative subgroup. All patients underwent an MRI examination. The inclusion criteria for the TN group were as follows: (1) TN diagnosis according to the International Classification of Headache Disorders (3rd Edition) (28); (2) treatment between October 2020 and December 2022 in the Department of Neurosurgery.

### 2.2 MR-DTI data acquisition, post-processing, and analysis

MR-DTI images [spin-echo planar imaging (EPI) sequence, *b*-value = 0 and 1,000 s/mm^2^, 72-direction, in-plane voxel size = 2 × 2 mm^2^, slice thickness = 2 mm, echo time (TE) = 95 milliseconds, repetition time (TR) = 4,100 milliseconds] were acquired with a 3.0 Tesla Siemens MR scanner (10, 16).

**Figure 1:**
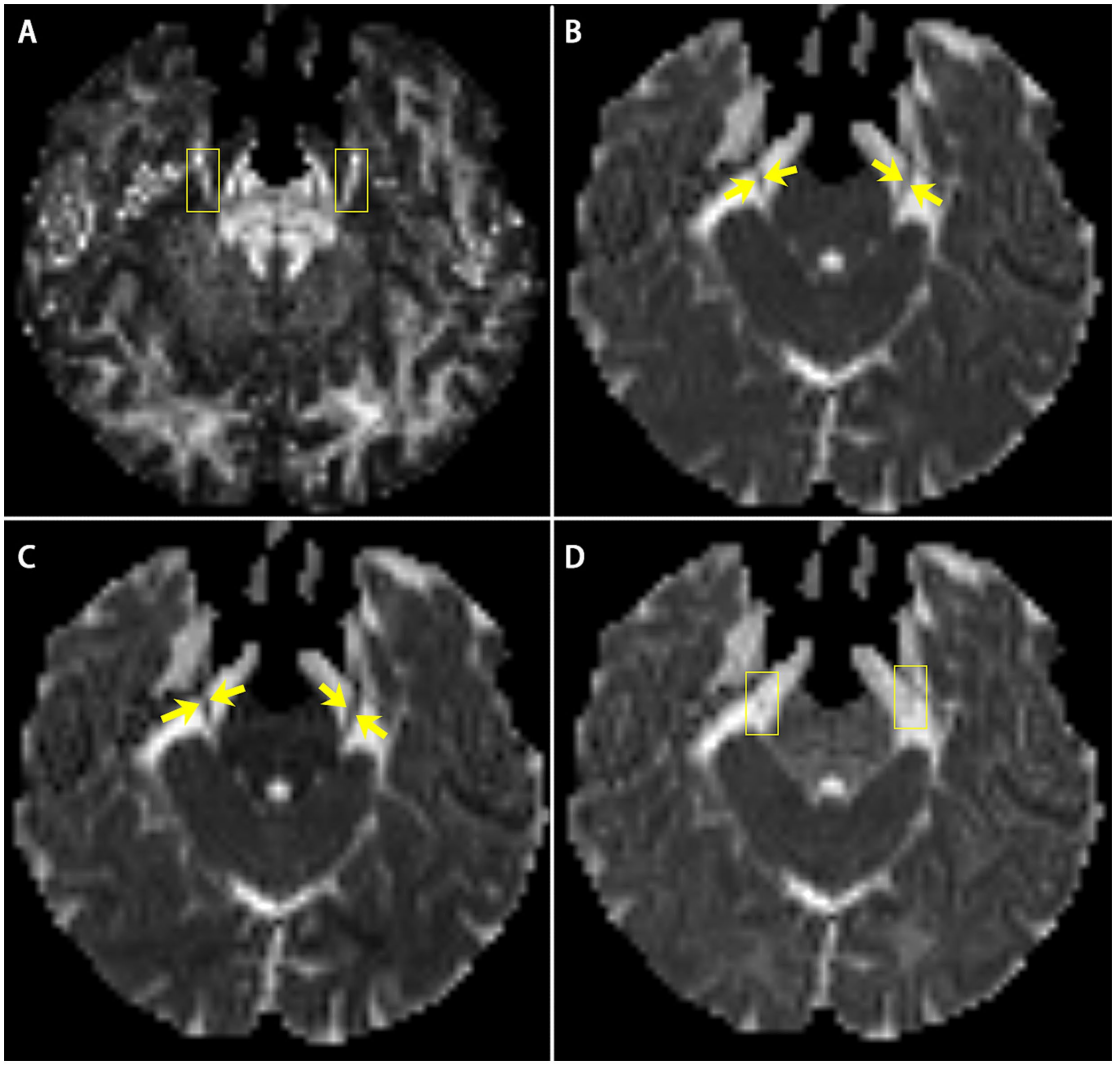
The scalar maps of four diffusivity metrics **(A)** FA; **(B)** MD; **(C)** AD; **(D)** RD. Yellow arrows and boxes in images show the bilateral CNV tracts in the scalar maps.

Image post-processing was performed applying 3D Slicer V4.11.^1^ The scalar maps of fractional anisotropy (FA), mean diffusivity (MD), radial diffusivity (RD), and axial diffusivity (AD) were calculated from raw data (Figure 1). CNV tracts were generated by tractography with seed points located at both sides of the CNV in FA maps (29, 30).

**Figure 2:**
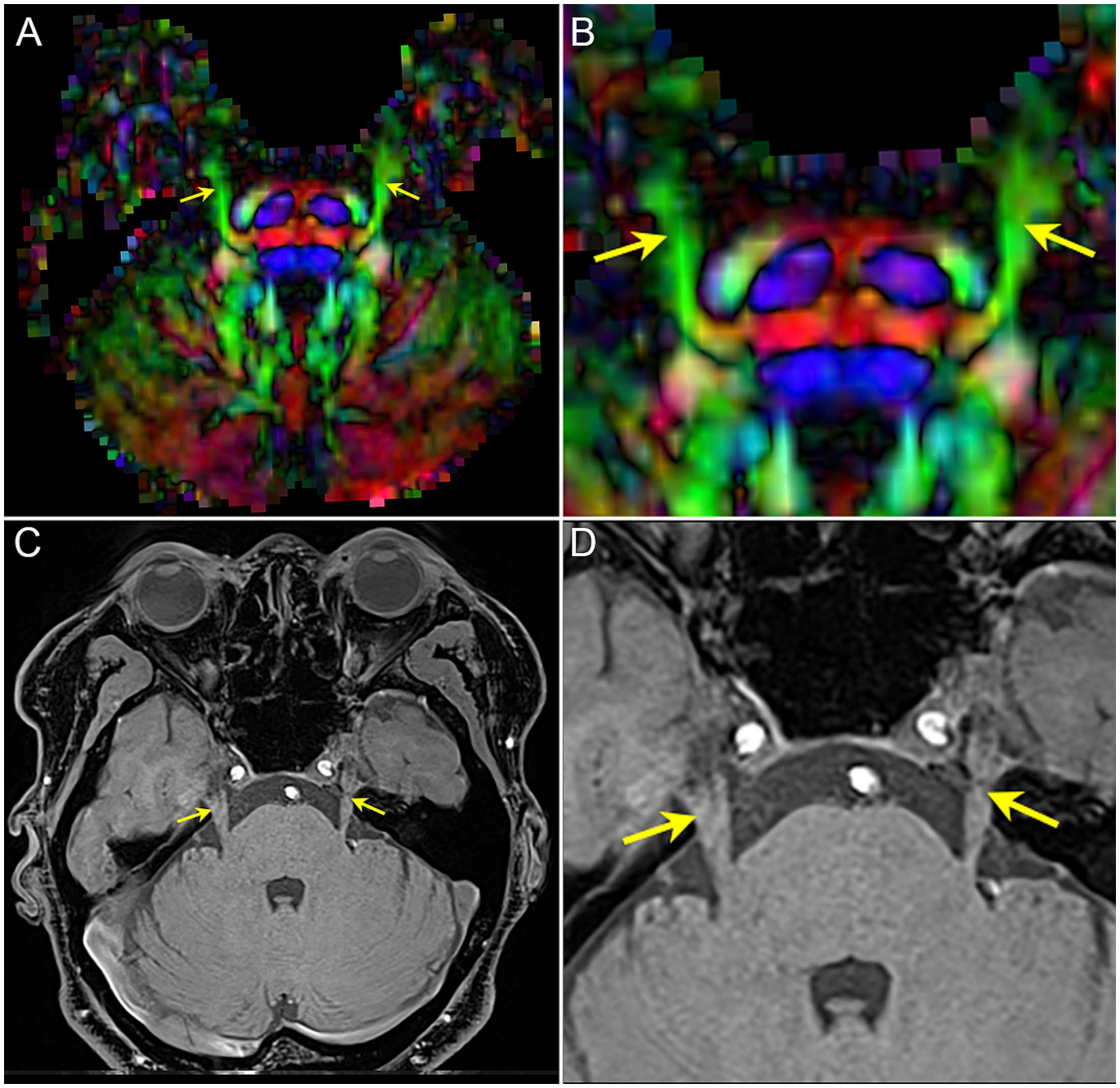
The image shows the bilateral CNV tracts in DTI and anatomical MRI. **(A,B)** the FA map of DTI image, image B is the focal magnification of **(A)**; **(C,D)** T1-weighted image of MRI, image D is the focal magnification of **(C)**. Yellow arrow: the bilateral CNV.

By three-dimensions images of DTI and MRI anatomical imaging, we identify the trigeminal ganglion area on the FA map (Figure 2). The TG is crescent-shaped, and the width of TG on axial position is between 3.58 and 8.19 mm (31–34). We selected 4 slicers (thickness 2.00 mm) to measure diffusivity metrics. Then, the FA values of the two-side TG are extracted from four consecutive slices on the coronal view of the FA map including the CNV tract. Regions of interest (ROI) were independently mapped in the same way by two trained personnel and subsequently confirmed by comparison to show reliability and avoid individual differences (35).

**Figure 3:**
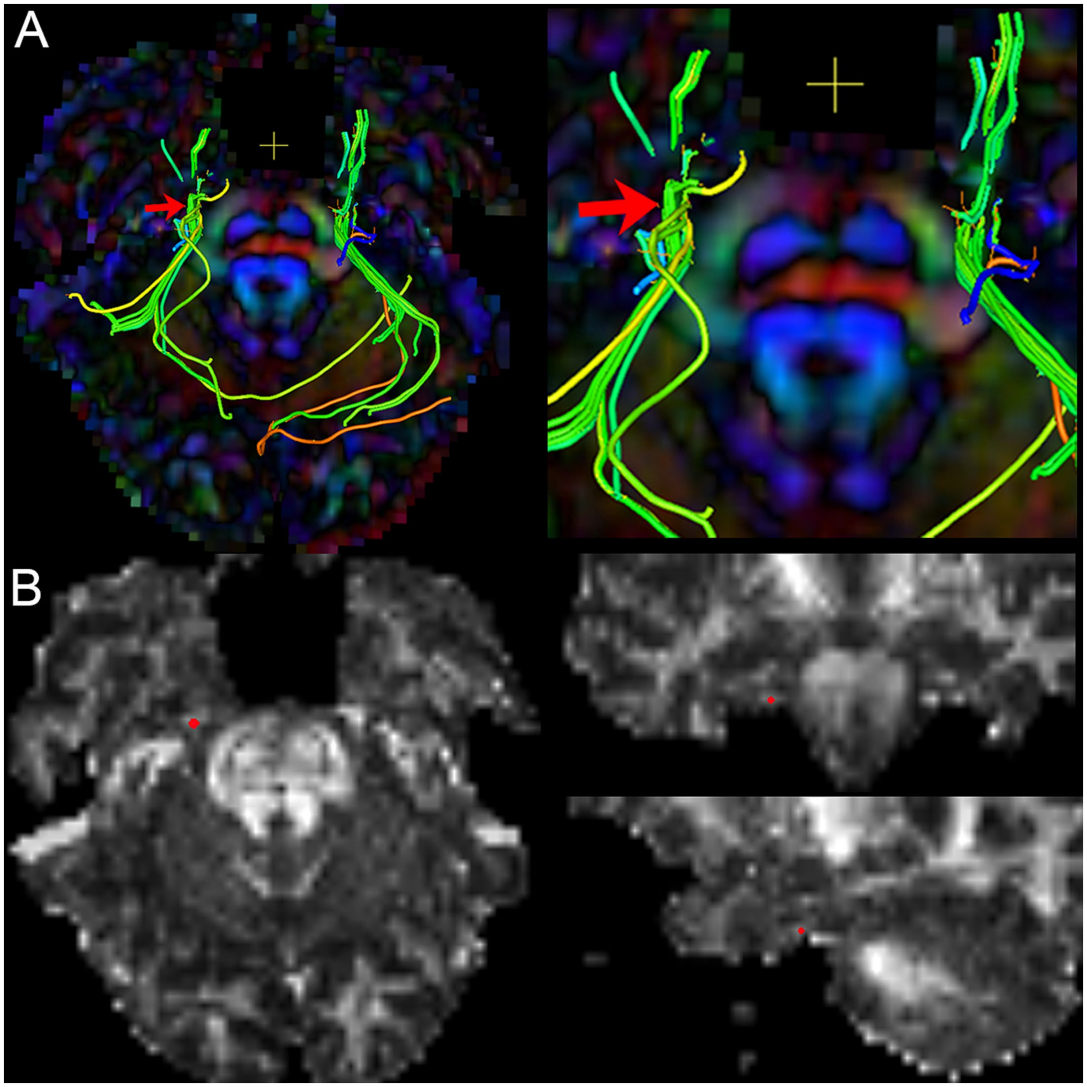
The image shows the extraction of diffusivity metrics on the bilateral CNV tracts. **(A)** the fused image of FA color-map and the reconstruction of CNV tracts, and image (right) is the focal magnification of image (left); **(B)** the position of the centroid on the CNV tract in axial, sagittal, and coronal view of FA map. Red arrow and red point: the position of the centroid.

To obtain the diffusivity metrics (FA, MD, AD, and RD) of CNV (Figure 3), the centroid was placed on the nerve tract in the successive coronal scalar maps and its position validated on the 3D planes and the CNV tracts. In total, 1–3 DTI scans (preoperative, postoperative, and follow-up) were performed in each TN patients.

### 2.3 Treatment

TN patients in the operative subgroup underwent PSR treatment. PSRs were performed using a stereotactic bidirectional approach-guiding technique (3, 7, 8) and guided by the position of the TGT on the TG in MR-DTI image (12). Meanwhile, TN patients in the nonoperative subgroup received medical treatment (carbamazepine or oxcarbazepine).

### 2.4 Treatment evaluation and follow-up

The treatment effect was evaluated according to the diffusivity metrics (FA, MD, RD, and AD) and using a visual analog scale (VAS) score postoperatively and during follow-up. The diffusion difference was evaluated by comparing the metrics between preoperative, postoperative, and follow-up. The follow-up time includes short-Term (3–6 months after PSR) and long-Term (18 months after PSR).

### 2.5 Statistical analysis

Statistical analysis was performed on all DTI indicators. The DTI metrics and pain scores were compared between preoperative and postoperative using the paired *t*-test. The correlation between diffusivity metrics and pain scores was calculated by Pearson correlation. All statistical analyses were performed using SPSS v26.0 (IBM, USA). In all statistical tests, *p* < 0.05 was considered statistically significant.

### 2.6 Ethical disclosure statement

This study involving human participants was in accordance with the ethical standards of the institutional and national research committee and with the 1964 Helsinki Declaration and its later amendments or comparable ethical standards. This study was approved by the Ethics Committee of the China-Japan Union Hospital of Jilin University, Approval no. 2023102704.

### 2.7 Informed consent

All participants provided an informed written consent. All patient-related data in this article were anonymized.

## 3 Results

### 3.1 Patient characteristics

The baseline characteristics of the 28 TN patients are summarized in Table 1. Overall, 15 patients (Patients 1–15) underwent PSR treatment. A total of 13 patients selected medical treatment.

**Table 1:** The characteristics of TN patients (*n* = 28).

Characteristic	Number of Patients
Total	28
Age and Sex	
Age range	38–85
Age (Mean ± SD)	62.8 ± 12.1
Male	14
Female	14
Affected side	
Right	19
Left	9
V1	1
V2	11
V3	5
V1 + V2	5
V2 + V3	3
V1 + V2 + V3	3

### 3.2 The manifestation of diffusivity metric on bilateral trigeminal ganglion in TN patients

We analyzed the data of diffusivity metrics on the bilateral trigeminal ganglion in 28 patients with TN. The result reveals a significant difference of FA between the affected and unaffected TG side with no significant changes of MD and AD.

### 3.3 The manifestation of diffusivity metric on bilateral trigeminal ganglion before and after treatment in PSR patients

**Figure 4:**
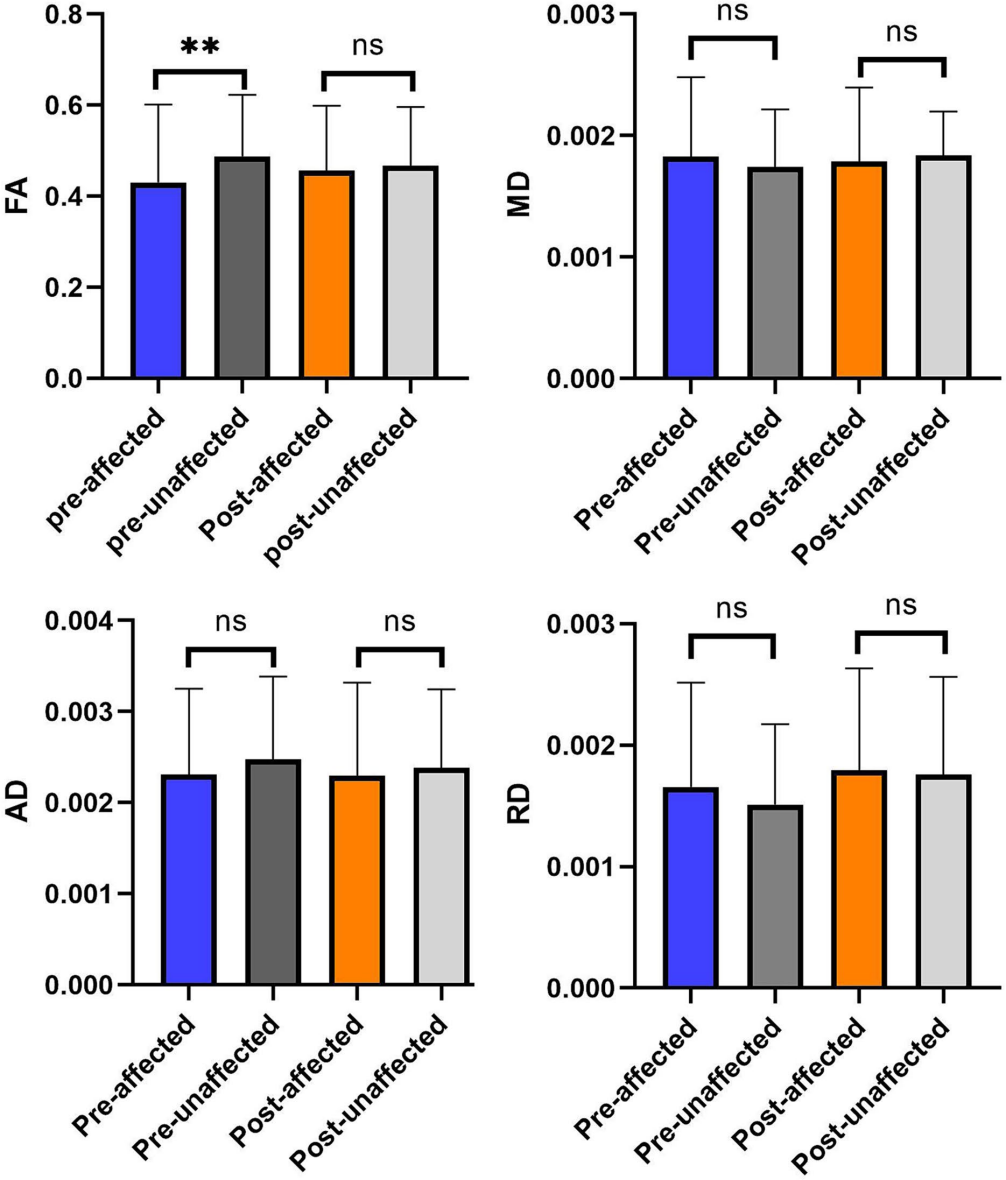
The comparison of diffusivity metrics on the CNV tracts between the affected and unaffected sides in preoperative and postoperative. **p < 0.01; ^ns^*p* > 0.05.

We analyzed the data of diffusivity metrics on the bilateral trigeminal ganglion in patients with PSR (Figure 4).

After the effective radiofrequency treatment, diffusivity metrics of the affected side TG and the unaffected side TG are similar. There is no significant difference in diffusivity metrics between the affected and unaffected sides in after the procedure (*p* > 0.05).

### 3.4 The longitudinal change of diffusivity metric on the affected or unaffected trigeminal ganglion during treatment

**Figure 5:**
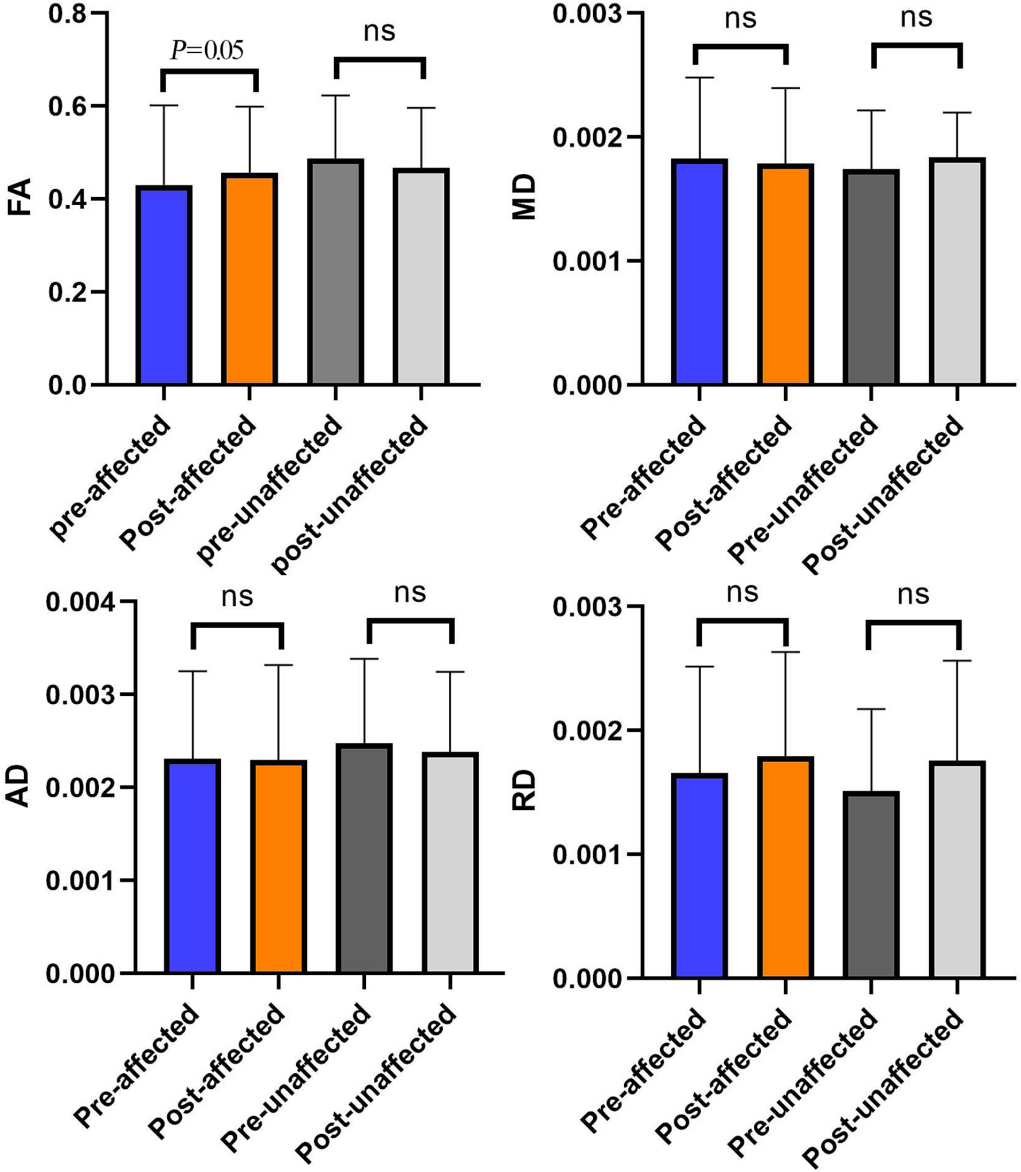
The comparison of diffusivity metrics on the bilateral CNV tracts between preoperative and postoperative. *p < 0.05; ^ns^*p* > 0.05.

We analyzed the comparison of diffusivity metrics on the trigeminal ganglion in PSR patients before and after treatment (Table 2 and Figure 5). By analyzing the data of diffusivity metrics on the affected side trigeminal ganglion, we found that the FA of the affected side trigeminal ganglion is significantly increased after treatment, and the RD of the affected TG is obviously increased after PSR. Other diffusivity metrics had no obvious changes. There is an obvious change in FA on the affected side trigeminal ganglion between preoperative and postoperative (*p* = 0.05).

**Table 2:** The FA of the trigeminal ganglion with trigeminal neuralgia at preoperative and postoperative.

	The affected side of TG		The unaffected side of TG	
	Preoperative	Postoperative	Preoperative	Postoperative
Mean	0.41	0.46	0.49	0.47
Standard deviation	0.18	0.14	0.13	0.13
*T*-test	*P* = 0.05		*p* = 0.14	

**Figure 6:**
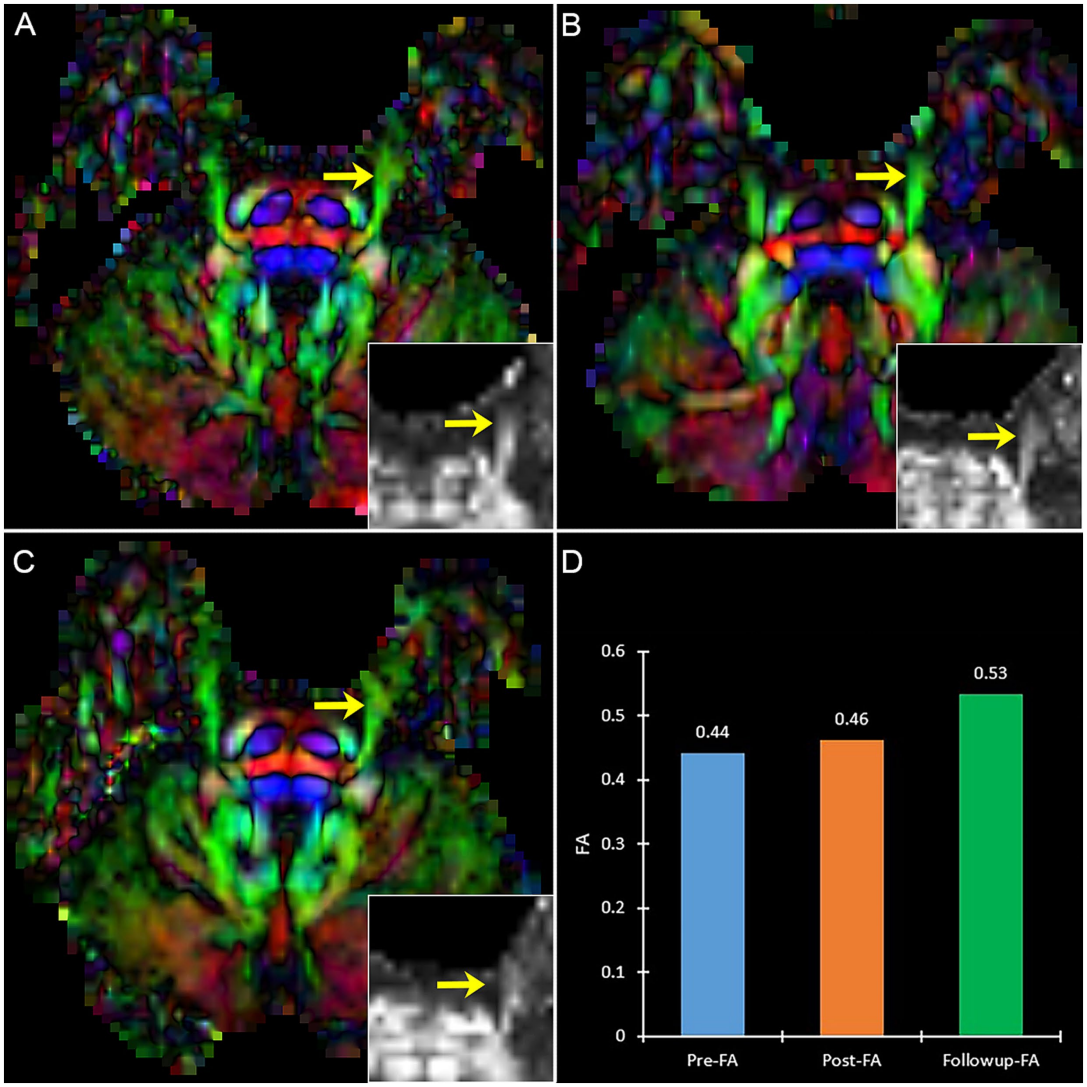
The DTI images (FA-color maps) of an effective patient after treatment in preoperative **(A)**, postoperative **(B)**, and follow-up **(C)**. Image **(D)** shows the FA values on three-time points. **(A–C)** show the bilateral CNV tracts, and the insert box is the local FA map on the affected CNV. Yellow arrow: the affected CNV tract.

**Figure 7:**
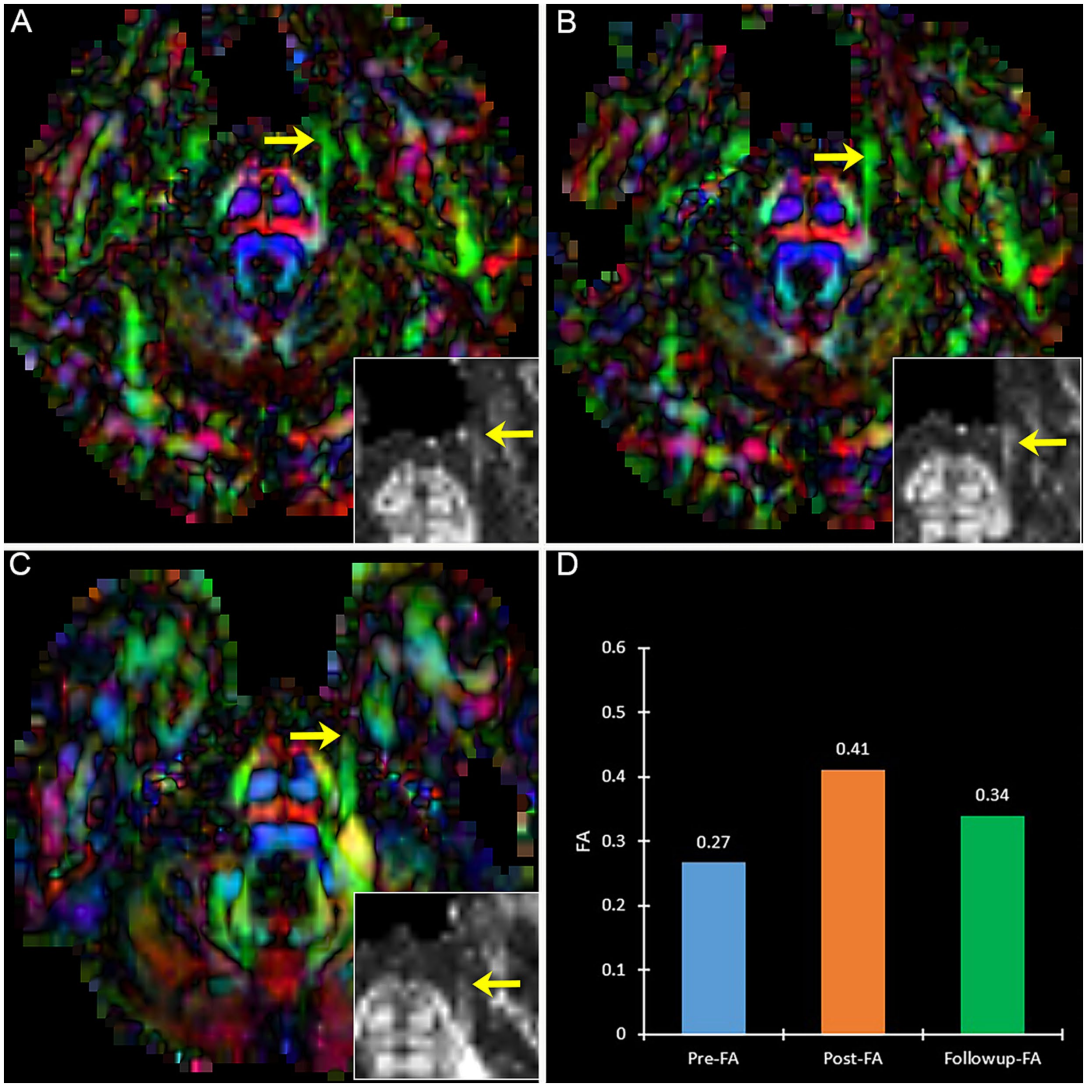
The DTI images (FA-color maps) of an ineffective patient after treatment in preoperative **(A)**, postoperative **(B)**, and follow-up **(C)**. image **(D)** shows the FA values on three-time points. **(A–C)** show the bilateral CNV tracts, and the insert box is the local FA map on the affected CNV. Yellow arrow: the affected CNV tract.

A patient with effective PSR treatment (Figure 6) manifests an increased trend of FA after treatment. Contrary to effective patients, a patient with ineffective PSR treatment in follow-up manifests a decreased trend of FA (Figure 7).

Meanwhile, the diffusivity metrics show the affected side TG and unaffected TG after treatment are approximately the same (*p* > 0.05).

### 3.5 Efficacy and complications

**Figure 8:**
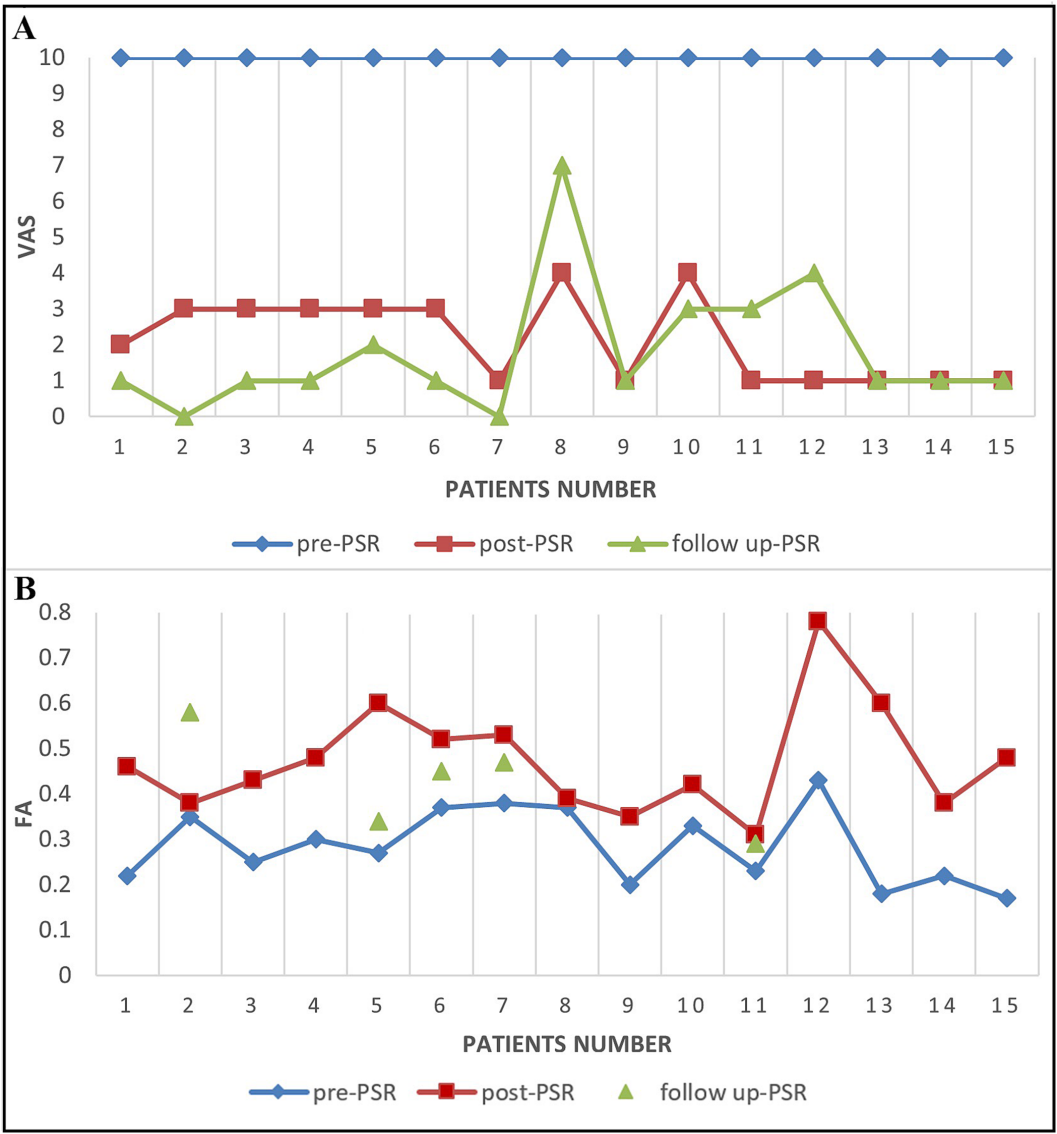
The VAS score and FA score changes in TN patients before and after PSR. **(A)** The VAS scores for patients before PSR (pre-PSR) and 2–3 days after PSR (post-PSR) and then 6 months after PSR (follow up-PSR). **(B)** The FA scores for patients before PSR (pre-PSR) and 2–3 days after PSR (post-PSR) and then 18 months after PSR (follow up-PSR).

VAS decreased postoperatively in 13 patients with VAS of 1–3 and 2 patients with VAS of 4 at 3-day postoperative (Figure 8). In all patients in the operation subgroup, the postoperative decrease of VAS ranged from 60 to 90%. The postoperative FA value increased to 14.1% (Patient 8), 33.8–81.8% in 9 patients, and 124.2–274.9% in three patients.

A total of 2–3 days after the procedure, two patients (Patients 8 and 10) with a postoperative VAS score of 4 also had increased FA value of the TGT (14.1% in patient 8 and 33.8% in patient 10). The percent of decrease in FA value of the TGT changed compared with the adjacent area from a 43% reduction before surgery to a 19.1% reduction after surgery in patient 8. The VAS decreased to 1 at 4-day postoperatively in patient 10 and at 14-day postoperatively in patient 8.

There were obvious alterations in FA between pre-operation and post-operation (Figure 5). This result showed that preoperative FA was changed significantly by PSR on TGs in the operation subgroup.

At follow-up, VAS further decreased to 0–2 after a 6-month follow-up in 11 patients, and others with VAS of 3–7 (Patient 8, 10, 11, and 12) (Figure 8). Five patients underwent DTI at the 3-6^th^ month follow-up. Four patients had further FA increases compared with preoperative values, ranging from 24.7 to 103.8%. One recurrent patient (Patient 11) experienced a decrease in their FA value on TG by 26.6% when compared to their 3-day postoperative FA value (Figure 8). At 18 - month follow - up, there were still only four patients with recurrence, and the VAS of these recurrence patients was 7, 4, 4, and 2 (Figure 8).

Common complications including facial numbness did not occur, and only one patient (patient 10) experienced a rare complication of temporary apnea.

### 3.6 Correlations between the VAS scores and the diffusivity metrics of the affected trigeminal ganglion

By analyzing the VAS scores and diffusivity metrics of TN patients before and after treatment, we found that the diffusivity metrics (FA) and the VAS scores all changed at the same time (Figure 8). The alteration of the diffusivity metrics FA and the change of VAS scores are consistent in most patients (71.4%, 10/14). The diffusivity metric (FA) on the lowest FA area of the CNV preoperative is significantly negatively correlated with the VAS scores (Coefficient of correlation = −0.66, *p* < 0.05).

## 4 Discussion

The value of FA in TN patients may be an indicator of pain, and we call this indicator FA score. To determine if the FA score is a good indicator of pain, one needs to compare the FA score to an accepted pain indicator, such as VAS. In the present study, the FA value is compared between affected and unaffected trigeminal ganglia. Our results suggest that FA can differentiate whether the patient has neuralgia but no other pain. The FA score may be an indicator of some damage or changes unrelated to pain but is still useful to diagnose trigeminal neuralgia.

DTI is an advanced neuroimage technique based on the principle of diffusion motion of water protons in nerve tissue, and it has recently received attention in TN. DTI was used to evaluate the efficacy of MVD and gamma knife radiosurgery (GKRS) with the therapeutic target in REZ for TN (21, 22, 36). However, DTI has rarely been reported to adequately identify the diffusion alteration on TG of radiofrequency treatment for TN (12). In our study, we investigated the sensitive diffusivity metric reflected by the microstructural alteration of TG during the treatment of TN, and explored the correlation between the treatment effect and the diffusivity metrics of TG.

As a comprehensive reflection of the diffusion profile, FA is the most widely used metric in the dominant diffusion direction. Diffusivity metrics, especially FA, reflect the treatment efficacy in the microstructure of TN patients (37). FA has a high sensitivity to the microstructure of myelinated axons and neural injury. FA has been reported to have high predictive value for the prognosis of patients who undergo radiofrequency treatment (9, 21). Several reports showed that FA could be used as a biomarker to evaluate the efficacy of MVD and GKRS (19, 38, 39). FA can normalize toward the level of control after effective treatments (17, 21, 40). A GKRS study reported a decrease in FA at the CNV target and that the recurrent pain was related to FA reversal (36). Thus, it is generally regarded as a quantitative, noninvasive treatment effect and prognostic marker of TN (9, 10, 23, 39, 41–44). Further, the efficacy of treatment was confirmed through postoperative and follow-up evaluations.

In our study, we found that the FA of the affected side TG was lower than that of the unaffected side TG preoperatively and increased postoperatively. The decreased FA preoperative reflected the nerve tissue injury, and the increased FA postoperative showed the treatment outcome of nerve injury. The injury of the CNV mainly is demyelination with lower FA in DTI. The obvious changes of the FA on the affected side after treatment suggested that the treatment altered the microstructure of TG. There is no significant difference in the FA of the two side TG after treatment and the preoperative significant difference in the two side TG has disappeared after treatment. The treatment target of PSR was only the TG. Therefore, the change of FA on the affected side TG in pre-and postoperative may be correlated with PSR to the TG and reflected the microstructural change of TG after PSR.

The correlations between diffusivity metrics and the VAS scores or treatment outcome suggested the significance of diffusivity metrics to evaluate the treatment effects (21, 36, 37, 45). All patients in the operation subgroup had decreased VAS postoperatively and at follow-up. Meanwhile, we use FA as a metric to evaluate the efficacy of PSR with TG. There was a significant increase in FA (*p* < 0.01) postoperatively on TG, and no significant difference in the unaffected CNV. The alteration between the diffusivity metric FA and the VAS scores is consistent in all patients. Therefore, the most significantly changing in diffusivity of the TG is induced by PSR, and the significant change in diffusivity metric reflected the change in TG microstructure after PSR. The results support that the diffusivity metric on TG could be a useful indicator in quantitively evaluating PSR efficacy and assessing pain intensity.

Furthermore, two patients (patients 10 and 8) had VAS of 4 at 3 days postoperatively, but their VAS decreased to 1 at postoperative days 4 (patient 10) and 14 (patient 8). The changes of FA on TG in a short time (2–3 days postoperative) are low FA value (patient 10) and decrease range of FA value (patient 8). Therefore, the treatment effect could be attributed to their change of FA postoperative. In comparison, VAS has been affected by subjective factors and emotion to some extent in the assessment of treatment effect. Whereas, diffusivity metrics are more objective to assess the treatment effect. The results support that the diffusivity metric FA on TG could be objective quantitative useful indicators in evaluating the PSR efficacy and predicting treatment outcomes (9, 46).

At present our study, the efficacy (pain recurrence) was consistent between short-term (6 months) and long-term (18 months) postoperative follow-up. This suggests that short-term postoperative recurrence is an important issue and that DTI is an important tool for quantitative assessment of short-term postoperative efficacy.

Traditionally, the treatment effect of trigeminal neuralgia is assessed by pain scores, such as VAS (Visual Analogue Scale) and NRS (Numerical Rating Scale), but these pain scores are all susceptible to the patient’s emotional and psychological factors, and cannot accurately reflect the treatment effect in sometime. DTI is an objective quantification of nerve tract imaging to quantitatively assess the microstructure of the trigeminal nerve, and the advantage of DTI is that it can quantitative comparisons of the microstructural parameters of the nerve tract before and after treatment are performed as a means of objectively and quantitatively assessing the treatment effect. However, there are some limitations of DTI compared to other pain assessment tools, including that DTI can only be applied in hospital which have appropriate MR equipment (e.g., 3.0 T MR) and with the ability to access the DTI images and the diffusion data in an operation team.

In brief, TGs with diffusivity metrics were beneficial for evaluating the treatment efficacy objectively, and predicting treatment outcomes.

## 5 Conclusion

Diffusion tensor imaging (DTI) is capable of investigating the longitudinal changes of FA on the trigeminal ganglion before and after radiofrequency treatment in TN patients, and diffusivity metrics could be an independent reliable efficacy indicator for TN. There is a correlation between the VAS and diffusivity metric of TG, and the alteration of the diffusivity on TG may be correlated with the effect of radiofrequency treatment. In addition, DTI may provide further insight into the prediction of treatment efficacy.

## 6 Limitations of the study

Some of the limitations of our study are as follows: One, a more reliable criterion for trigeminal ganglion target (TGT) needs to be established in larger studies. Second, the applicability of TGT in TN patients is unclear and needs to be further verified and follow-up.

## Data Availability

The original contributions presented in the study are included in the article/supplementary material, further inquiries can be directed to the corresponding authors.

## Footnotes

^1^ http://www.slicer.org/
